# Microvascular dysfunction and heart failure with preserved ejection fraction

**DOI:** 10.1007/s00392-025-02822-1

**Published:** 2026-01-08

**Authors:** Ornela Velollari, Antonio Biancofiore, Maximilian Olschewski, Helen Ullrich-Daub, Thomas Münzel, Karl-Philipp Rommel, Philipp Lurz, Tommaso Gori, Karl-Patrik Kresoja

**Affiliations:** 1 Department of Cardiology, Cardiology I, University Medical Center Mainz, Mainz, Germany; 2 German Centre for Cardiovascular Research (DZHK), Standort RheinMain, Mainz, Germany; 3 Department of Interventional Cardiology, Misericordia Hospital, Azienda USL (Unità Sanitaria Locale) Toscana Sud Est, Grosseto, Italy

**Keywords:** Coronary microvascular dysfunction, Diastolic dysfunction, Heart failure with preserved ejection fraction

## Abstract

**Background and aim:**

The impact of coronary microvascular dysfunction (CMD) on parameters of systolic and diastolic function, particularly in patients with heart failure and preserved ejection fraction (HFpEF), is poorly understood. Although these conditions often overlap, their combined impact on parameters of systolic and diastolic function remains unexplored.

**Methods:**

Consecutive patients undergoing invasive CMD assessment were enrolled. Systolic function was assessed by global longitudinal strain (GLS), diastolic function by E/E’, left atrial reservoir strain (LARS), and non-invasive single-beat pressure–volume relationship stiffness constant (β) estimation.

**Results:**

Of 145 patients, 72 (49.7%) had CMD, 35 (24%) HFpEF, and 23 (16%) both conditions. HFpEF was twice as prevalent in CMD patients as compared to patients without CMD (32% vs. 16%, *p* = 0.034). Both CMD was associated with parameters of both diastolic (E/E’ β = 0.29, *p* < 0.001, LV stiffness constant β = 0.23; *p* = 0.006, and LARS β = -0.21, *p* = 0.02) and systolic function (GLS β = 0.31, *p* < 0.001). The presence of CMD was associated with an impairment in LV stiffness both in patients with and without HFpEF (p for interaction = 0.044).

**Conclusion:**

HFpEF is highly prevalent in patients with CMD, and CMD is associated with both systolic and diastolic dysfunction. In patients with HFpEF, an additional worsening of diastolic function is observed.

**Graphical Abstract:**

**Figure:**
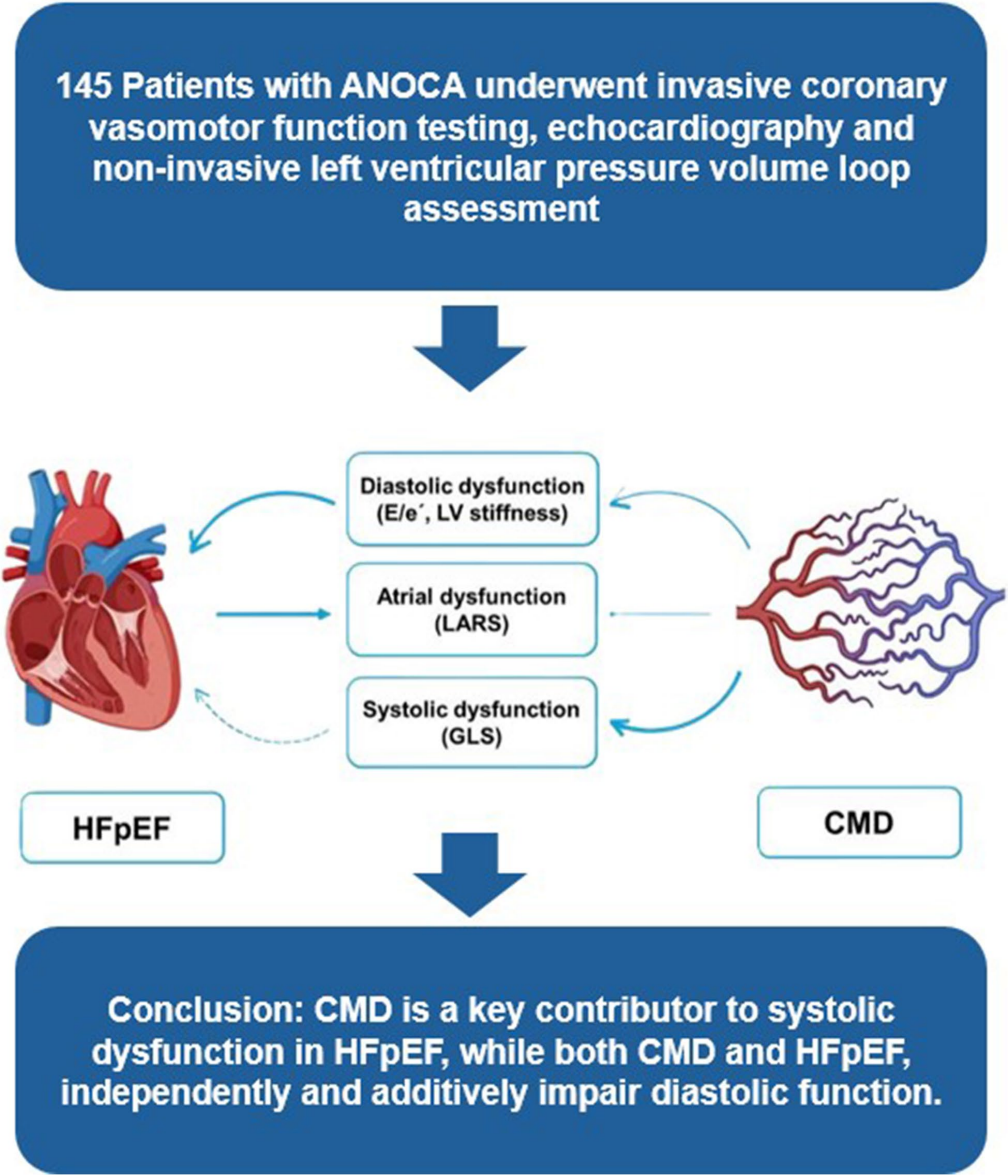

## Introduction

Coronary microvascular dysfunction (CMD) is a frequent condition with important implications for patients’ outcomes and quality of life, particularly in patients with heart failure and preserved ejection fraction (HFpEF) [1, 2].

CMD is a multifactorial condition caused by structural (capillary rarefaction, compression, obstruction, remodeling) and/or functional phenomena (impaired vasomotion), ultimately leading to ischemia. The resulting impairment in myocardial perfusion may cause symptoms, and it might impair both systolic and diastolic function [3–5]. In turn, the increased wall tension associated with increased left ventricular end-diastolic pressure (LVEDP) in HF might reduce microvascular compliance, resulting in CMD. Therefore, CMD and HFpEF as distinct entities may interact bidirectionally, although the extent of the pathophysiological overlap between CMD and HF remains incompletely understood. In patients with HFpEF, non-invasively measured impairments in coronary flow reserve (CFR) have been associated with elevated markers of congestion, more severe diastolic and systolic dysfunction, impaired left atrial function, higher prevalence of atrial fibrillation, and higher hospitalization rates [6, 7].

The present study aimed to determine the prevalence of HFpEF in a cohort of patients undergoing gold-standard invasive testing for CMD and to explore the independent and combined associations of HFpEF and CMD with markers of systolic and diastolic function.

## Methods

### Inclusion and exclusion criteria

Consecutive patients with symptoms of angina (CCS II–IV) and no relevant coronary obstruction were enrolled in a prospective, observational study (MICRO trial: NCT04612322).

Specific inclusion criteria were: age 18–85 years, presence of sinus rhythm, exclusion of epicardial stenoses (defined as FFR > 0.80). Patients with severe kidney disease (eGFR < 30 ml/min/1.73 m^2^), known cardiomyopathies, reduced LVEF, severe valvulopathies, epicardial, and microvascular coronary spasm during acetylcholine provocation testing were excluded from the present analysis. Patients with stable symptoms and without signs of decompensated heart failure or acute heart failure were included, thereby representing a cohort with early HFpEF phenotype. This approach allowed for the assessment of CMD without the confounding effects of advanced structural heart disease.

All study procedures were performed in accordance with the Declaration of Helsinki and were approved by the local ethics committee. All participants provided written informed consent.

### Definitions

HFpEF was defined retrospectively according to the 2016 European Society of Cardiology diagnostic algorithm, where three criteria had to be fulfilled: 1. BNP (B-type natriuretic peptide) elevation ≥ 35 pg/ml; 2. LVEF (left ventricular ejection fraction) ≥ 50% and NYHA class (New York Heart Association) > I; 3. evidence of diastolic dysfunction on echocardiography: E/E’ > 13; lateral or septal E’ < 9 cm/s; LAVI (Left atrial volume index) > 34 ml/m^2^ or LVMI (Left ventricular mass index) for women > 95 g/m^2^ and men > 115 g/m^2^ [8]. CMD was defined as reduced coronary flow reserve (CFR ≤ 2.5) and/or elevated index of microvascular resistances (IMR ≥ 25) [9] following maximal hyperemia induced by adenosine.

### Cardiac catheterization and microvascular function assessment protocol

Invasive assessments were conducted according to a standardized protocol [10] (Supplementary methods). Epicardial/microvascular spasm was diagnosed in the presence of angiographic obstruction, angina pectoris, and ischemia ECG changes during acetylcholine administration as per Coronary Vasomotion Disorders International Study Group (COVADIS) criteria [11].

### Echocardiographic assessment

Two-dimensional transthoracic echocardiographic analysis was performed with Philips ultrasound devices (Philips Intera, Philips Healthcare, Amsterdam, The Netherlands). Mitral valve inflow pattern (E and A velocity), septal and lateral mitral valve annular velocities (E’), as well as pulmonary arterial systolic pressure (PAPs), left atrial area (LAA), isovolumetric relaxation time (IVRT), deceleration time (DT), end-diastolic left ventricular volume (LVEDV), LAVI, and LVMI were recorded in an apical 4-chamber view. The ratio of E/A, septal E/E’, lateral E/E’, average E/E’, PAPs, LAVI, and LVMI was calculated as markers of diastolic function according to recommendations from the American Society of Echocardiography and the European Association of Cardiovascular Imaging [12]. Speckle tracking analysis of 2D echocardiographic images was performed using Philips QLAB software (Philips Intera, Philips Healthcare, Amsterdam, The Netherlands). Left ventricular global longitudinal strain (GLS) was measured across three apical views (four-chamber, two-chamber, and three-chamber), while left atrial reservoir strain (LARS) was assessed in the apical four-chamber and two-chamber views. Both GLS and LARS were represented as the average value of all conducted measurements.

Pressure–volume estimations were assessed non-invasively using a single-beat estimation method, as proposed by Klotz et al. [13] for the end-diastolic pressure–volume relationship. Further details into single-beat estimations are outlined in the supplemental material. The stiffness constant β was used as the primary readout of the non-invasive pressure volume loop derived diastolic function.

### Statistical analysis

Continuous variables were tested for parametric distribution using the Kolmogorov–Smirnov test. Normally distributed variables are presented as mean ± standard deviation, whereas non-normally distributed variables as median[interquartile range]. Categorical variables are displayed as frequencies and percentages. Fisher's exact test was used to compare categorical data between groups. The Kruskal–Wallis test was used to compare continuous data with non-parametric distribution and unpaired Student's t-test to compare variables with parametric distribution. Linear regression analysis – first univariable, then multivariable analysis – was performed to identify correlation between CMD, as well as HFpEF with E/E’, LARS, GLS, and the stiffness constant β. To account for different scaling of variables, the standardized regression coefficient β* is reported for comparison between groups and variables. Interaction p-values were calculated to evaluate between-subject effects. Lastly, receiver-operating characteristic curve (ROC) analysis was performed, with estimation of Youden index-derived cut-off value for a given predictor variable of CMD in HFpEF patients. Statistical analysis was performed with IBM SPSS Statistics software, Version 29, Armonk, NY: IBM Corp. A two-tailed P-value < 0.05 was considered statistically significant.

## Results

### Patient characteristics

**Fig. 1:**
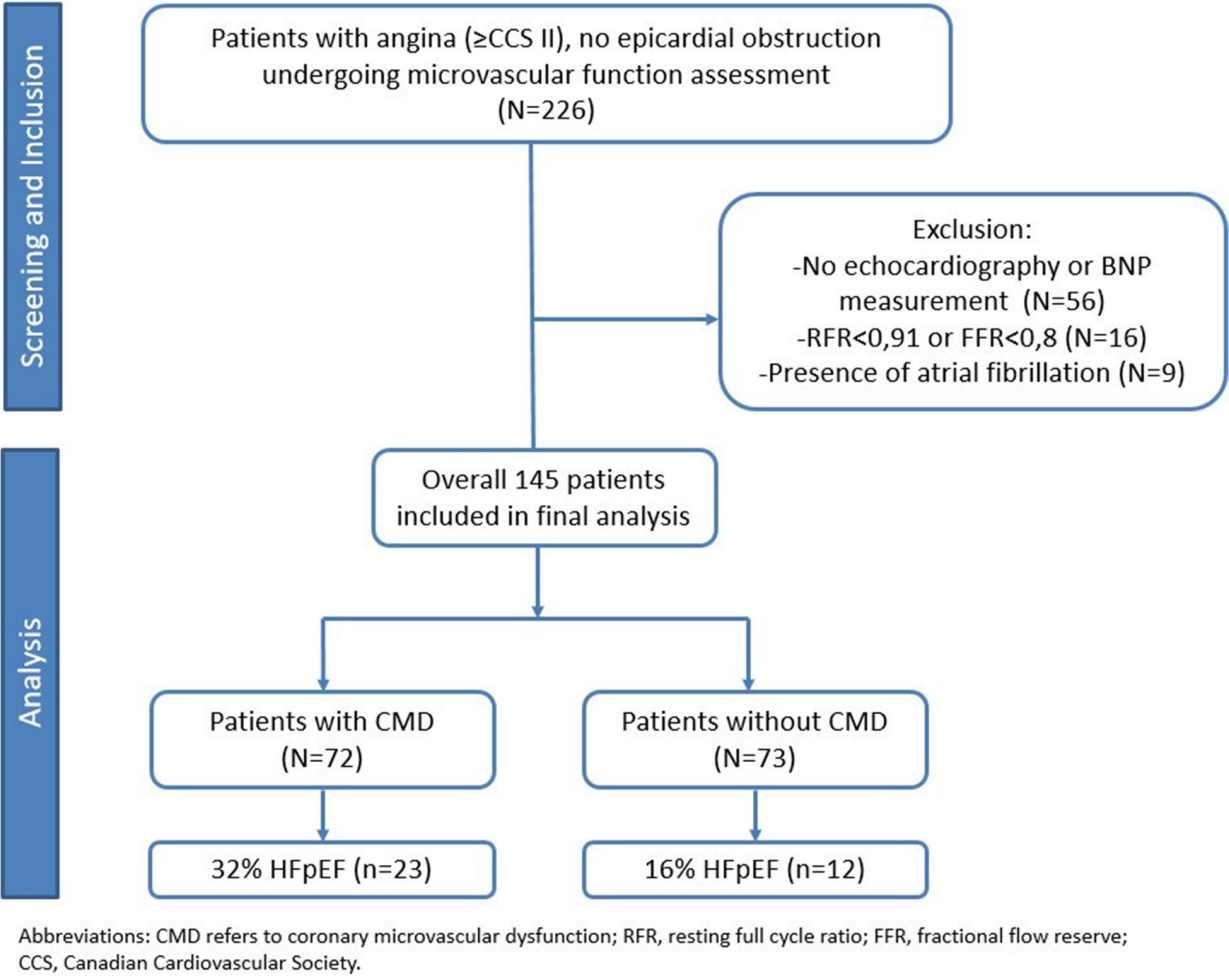
Flow chart illustrating the selection of patients in the study

One hundred and forty-five patients were included between February 2019 and October 2022 (Fig. 1). Overall, 72 patients (49.7%) had CMD, and 35 patients (24%) had HFpEF. HFpEF was more frequent in patients with CMD (23/72, 32%) compared to those without (12/73, 16%, *p* = 0.034).

The baseline characteristics of the patients are shown in Table 1. Patients with combined HFpEF and CMD were older (80 [70–83] years; *p* < 0.001), had a higher prevalence of diabetes (6/23, 26%; *p* = 0.03), displayed a higher concentration of BNP (pg/ml) (129 [84–166]; *p* < 0.001), showed LA enlargement (24 [21–28] cm^2^; *p* < 0.001), higher LV stiffness constants ß (6.3 [6.1–6.6]l/min; *p* < 0.001), lower stroke volumes (46 [39–53]ml; *p* = 0.02), LV end-diastolic volumes (81[71–93]ml; *p* = 0.01) and LARS (32 [15–30]%; *p* = 0.001), and higher systolic pulmonary artery pressures (sPAP) (33 [28–38]mmHg; *p* < 0.001).

**Table 1:** Baseline, echocardiographic, and angiographic characteristics for HFpEF/CMD subgroups

	CMD +/HFpEF + (*N* = 23)	CMD +/HFpEF- (*N* = 49)	CMD-/HFpEF + (*N* = 12)	CMD-/HFpEF- (*N* = 61)	*P*-value
Baseline characteristics					
Age, y	80 [70–83]	64 [58–76]	69 [60–77]	61 [57–71]	< 0.001*
Male sex, n (%)	7 (30%)	20 (41%)	6 (50%)	31 (52%)	0.36
Hypertension, n (%)	22 (96%)	39 (81%)	11 (92%)	46 (78%)	0.23
CCS, n (%)					0.43
*I*	1 (6%)	2 (5%)	1 (12.5%)	1 (2.5%)	
*II*	6 (38%)	18 (46%)	6 (75%)	14 (36%)	
*III*	7 (44%)	17 (44%)	1 (12.5%)	20 (51%)	
*IV*	2 (13%)	2 (5%)	0 (0%)	4 (10.5%)	
NYHA, n (%)					0.69
*I*	12 (55%)	32 (65%)	9 (75%)	36 (63%)	
*II*	9 (41%)	17 (35%)	3 (25%)	19 (33%)	
*III*	1 (4%)	0 (0%)	0 (0%)	0 (0%)	
*IV*	0 (0%)	0 (0%)	0 (0%)	1 (2%)	
Dyslipidemia, n (%)	15 (65%)	35 (71%)	6 (50%)	35 (57%)	0.35
Diabetes mellitus, n (%)	6 (26%)	9 (18%)	1 (8%)	3 (5%)	0.03*
Tobacco exposure, n (%)	12 (52%)	24 (49%)	5 (42%)	26 (43%)	0.84
Obesity, n (%)	17 (77%)	40 (82%)	8 (67%)	36 (59%)	0.06
BMI (kg/m^2^)	27 [25–29]	28 [25–31]	27 [25–30]	26 [24–29]	0.24
BNP, pg/ml	129 [84–166]	22 [11–34]	70 [45–109]	19 [10–36]	< 0.001*
HFpEF, n (%)	23 (100%)	0 (0%)	12(100%)	0 (0%)	< 0.001*
HFA-PEFF-Score	4.4 ± 1.2	1.5 ± 1.2	3.4 ± 1.4	1.1 ± 1.2	< 0.001*
†H2FPEF-Score	3.9 ± 1.7	2.8 ± 1.4	3.7 ± 2.1	1.9 ± 1.4	< 0.001*
Echocardiographic characteristics					
LAA, (cm^2^)	24 [21–28]	19 [17–21]	20 [19–21]	17 [15–20]	< 0.001*
E/A	1.2 [0.7–1.7]	0.9 [0.7–1.1]	1.0 [0.7–1.3]	0.9 [0.8–1.2]	0.45
E/E’	13 [10–16]	9 [8–11]	9.4 [8.6–11]	8 [6.5–9]	< 0.001*
GLS, (%)	−17 [−20;−16]	−18 [−21;−16]	−19 [−21;−18]	−20 [−22;−19]	< 0.001*
LARS, (%)	21 [15–30]	30 [24–36]	25 [21–35]	34 [25–41]	0.001*
LAVi, (mL/m^2^)	37 [28–49]	28 [20–31]	39 [34–44]	28 [22–32]	< 0.001*
PASP (mmHg)	33 [28–38]	30 [26–34]	29 [24–34]	26 [24–30]	0.002*
IVS (mm)	12 [11–13]	11 [10–12.4]	11.2 [10–12]	11 [10–12.1]	0.42
ED LVV (ml)	81 [71–93]	82 [73–95]	100 [84–115]	90 [79–101]	0.01*
LVMI (g/m^2^)	78 [73–88]	66 [56–87]	65 [53–105]	72 [60–80]	0.18
LVEF (%)	56.1 ± 2.0	55.6 ± 2.3	54.7 ± 1.2	56 ± 2.5	0.20
Angiographic characteristics					
RFR	0.95 [0.93–0.97]	0.94 [0.92–0.96]	0.95 [0.91–0.97]	0.94 [0.91–0.96]	0.39
Resting flow (1/s)	1.5 [0.9–2.6]	1.5 [1.1–2.9]	2.3 [1.2–2.9]	1.8 [1.1–2.8]	0.69
Hyperemic flow (1/s)	3.1 [2.2–5.4]	3.3 [2.4–4.3]	11 [6–16]	8 [5–11]	< 0.001*
FFR	0.93 [0.88–0.96]	0.91 [0.88–0.94]	0.90 [0.85–0.94]	0.89 [0.85–0.93]	0.09
Ad-CFR	2.3 [1.6–2.8]	2.0 [1.4–2.7]	5.0 [4.0–6.3]	4.3 [3.0–5.8]	< 0.001*
Resting IMR (U)	65 [33–106]	66 [28–88]	40 [28–81]	54 [30–82]	0.67
Ad-IMR (U)	28 [15–49]	27 [20–34]	8 [5–12]	10 [7–15]	< 0.001*
Ad-MRR	2.5 [1.8–3.2]	2.5 [1.6–3.1]	6.0 [4.6–7.7]	5.0 [4.0–6.8]	< 0.001*
Ad-RRR	2.5 [1.7–3.1]	2.4 [1.6–3.0]	7.5 [5.0–11]	4.9 [3.7–6.5]	< 0.001*
Non-invasive end-diastolic pressure–volume loop relationship of the left ventricle					
SV (mL)	46 [39–53]	46 [40–52]	55 [46–63]	51 [44–57]	0.02*
LV stiffness constant β, (1/ml)	6.3 [6.1–6.6]	6.1 [6.0–6.2]	6.13 [6.1–6.3]	6.1 [6.0–6.11]	< 0.001*

*TA* = late diastolic transmitral flow velocity; *Ad* = Adenosine; *BMI* = Body mass index; *BNP* = B-type natriuretic peptide; *CCS* = Canadian Cardiovascular Society; *CMD* = Coronary microvascular dysfunction; *CFR* = Coronary flow reserve; *E* = early diastolic transmitral flow velocity; *E*` = early diastolic mitral annular velocity; *EDV* = End-diastolic volume; *FFR* = Fractional flow reserve; *GLS* = Global longitudinal strain; *HFpEF* = Heart failure with preserved ejection fraction; *IMR* = Index of microvascular resistance

### Association of CMD with systolic and diastolic function parameters

**Fig. 2:**
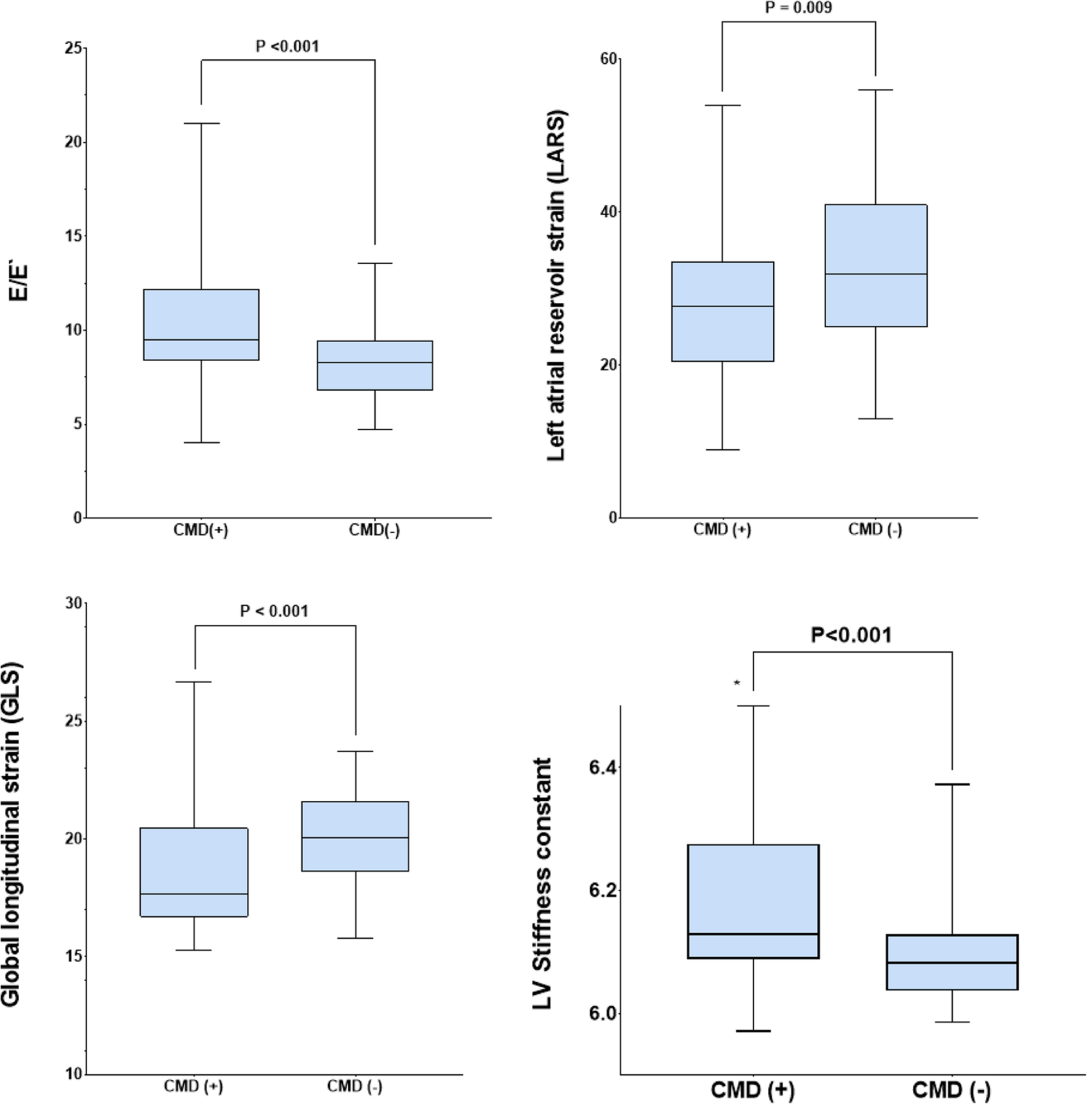
Changes in GLS, E/E’, LARS, and LV stiffness constant ß between CMD and control subjects. Box plots show median values of echocardiographic variables (GLS, E/E’, LARS, and LV stiffness constant ß) between CMD and control subjects, along with interquartile (25% to 75%) ranges

**Fig. 3:**
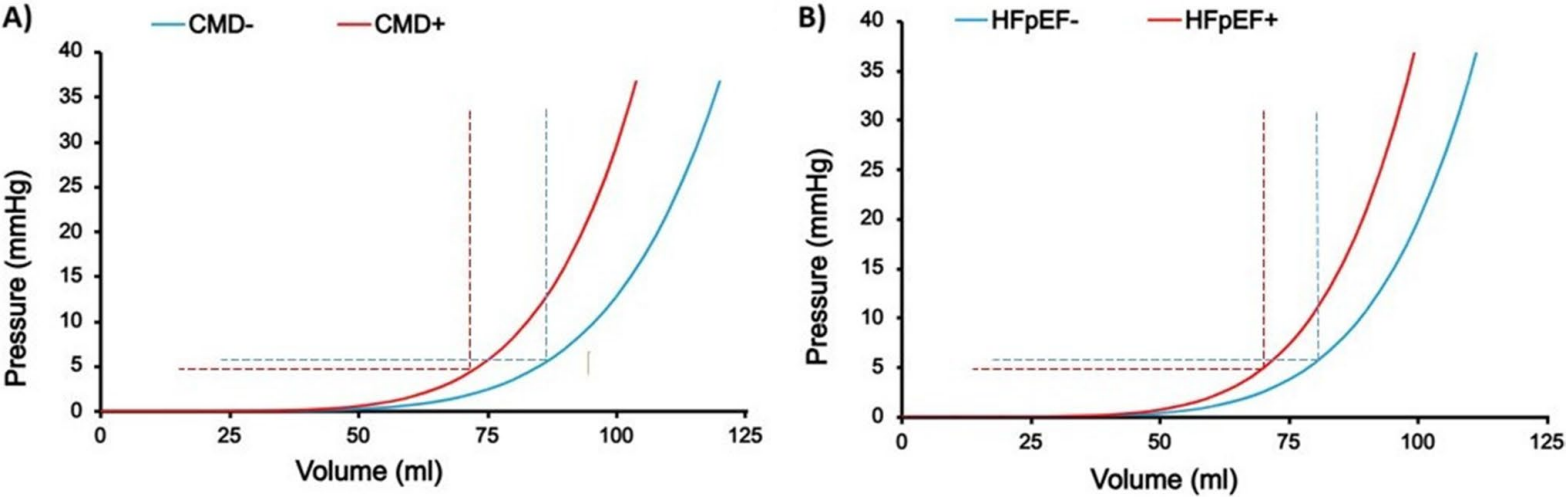
**A** Left ventricular end-diastolic pressure–volume relationship (LVEDPVR) curve for CMD and control subjects. EDPVR curve of patients with CMD (red line) displays a left and upwards shift compared to control subjects (blue line), indicating an increased LV stiffness and impairment of diastolic function. **B** Left ventricular end-diastolic pressure–volume relationship (LVEDPVR) curve for HFpEF and non-HFpEF patients. EDPVR curve of patients with HFpEF (red line) displays a left and upwards shift compared to non-HFpEF patients (blue line), indicating an increased LV stiffness and impairment of diastolic function

Patients with CMD had lower GLS and LARS, as well as higher E/e’ and LV stiffness constant β (Fig. 2). Accordingly, they displayed a significant association with GLS (β* = 0.34; *p* < 0.001), E/E’ (β* = 0.39; *p* < 0.001), LV stiffness constant β (β* = 0.31; *p* < 0.001), and LARS (β* = −0.27; *p* = 0.003). This remained significant for GLS, E/E’, and LV stiffness constant after adjustment for potential confounders such as age, diabetes, and obesity (Table 2). Further, patients with CMD displayed a left and upwards shift of the end-diastolic pressure–volume relationship (EDPVR) curve when compared to patients without CMD, implying a reduction of load-independent left ventricular diastolic function, similar to HFpEF patients (Fig. 3A/B).

**Table 2:** Univariable and multivariable regression analysis adjusted for confounder variables

Variable	GLS			LARS			E/E’			LV stiffness constant		
	Standardized Beta	Regression Coefficient ± SE	*p*-value	Standardized Beta	Regression Coefficient ± SE	*p*-value	Standardized Beta	Regression Coefficient ± SE	*p*-value	Standardized Beta	Regression Coefficient ± SE	*p*-value
Univariable regression analysis												
CMD	0.34	0.08 ± 0.02	< 0.001*	−0.27	−0.01 ± 0.004	0.003*	0.39	0.07 ± 0.02	< 0.001*	0.31	0.7 ± 0.2	< 0.001*
HFpEF	0.20	0.04 ± 0.02	0.02*	−0.33	−0.01 ± 0.003	< 0.001*	0.50	0.08 ± 0.01	< 0.001*	0.45	0.85 ± 0.15	< 0.001*
Multivariable regression analysis												
CMD*	0.27	0.06 ± 0.02	0.002*	−0.18	−0.008 ± 0.004	0.06	0.25	0.05 ± 0.02	0.006*	0.19	0.42 ± 0.20	0.03*
HFpEF*	0.13	0.02 ± 0.01	0.14	−0.25	−0.009 ± 0.003	0.01*	0.43	0.07 ± 0.01	< 0.001*	0.38	0.71 ± 0.16	< 0.001*

Dependent variables: GLS, LARS, E/E’ and LV stiffness constant

^*^Covariates: age, diabetes mellitus, and obesity

### Interplay of CMD and HFpEF with systolic and diastolic function parameters

**Fig. 4:**
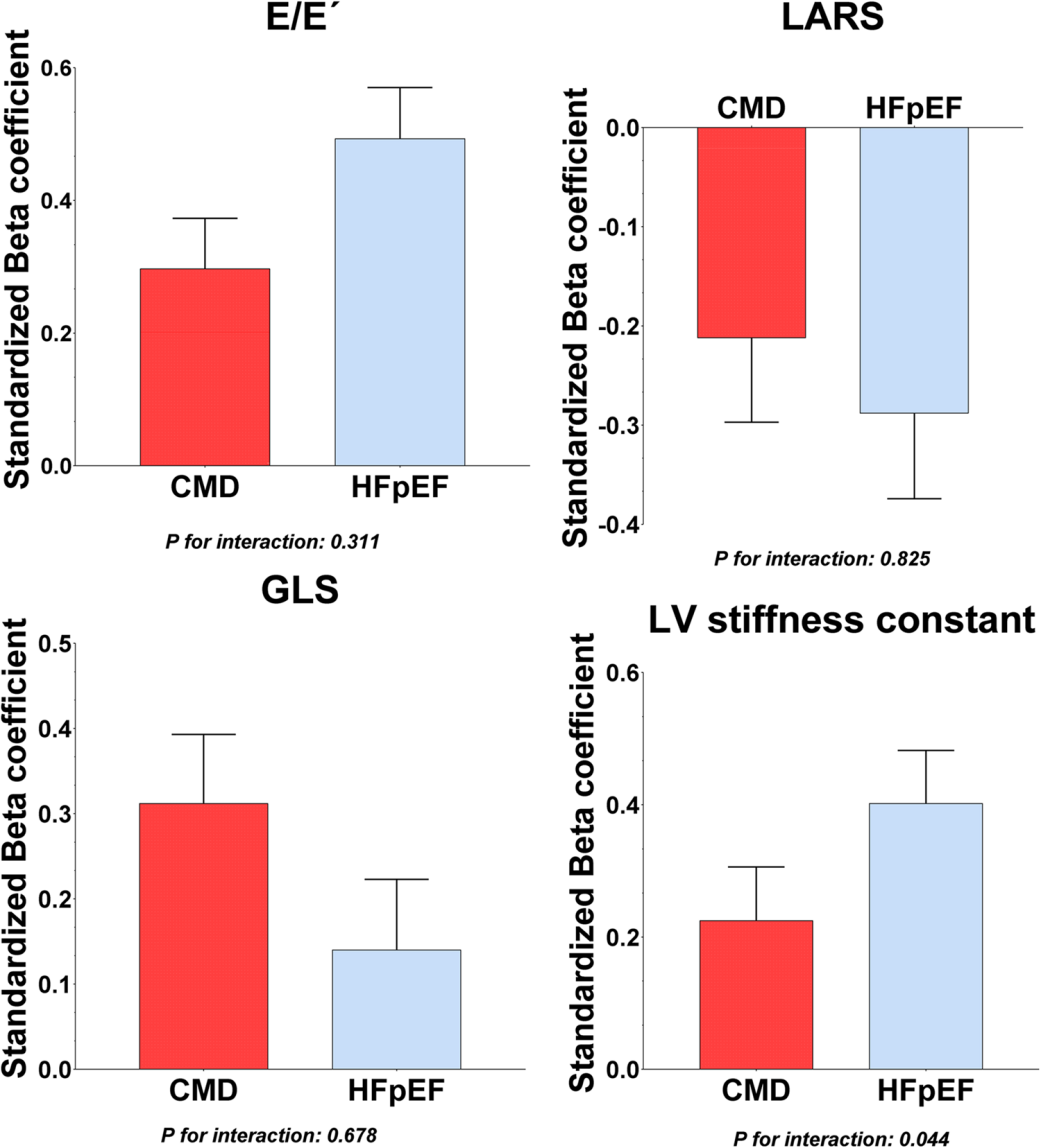
Comparison of influence between CMD and HFpEF on GLS, E/E’, LARS, and LV stiffness constant ß. Bar plot diagrams depict standardized regression coefficients (β*) for CMD and HFpEF and dependent echocardiographic variables (GLS, LARS, E/E’, and LV stiffness coefficient), with corresponding *p*-values for interaction

**Fig. 5:**
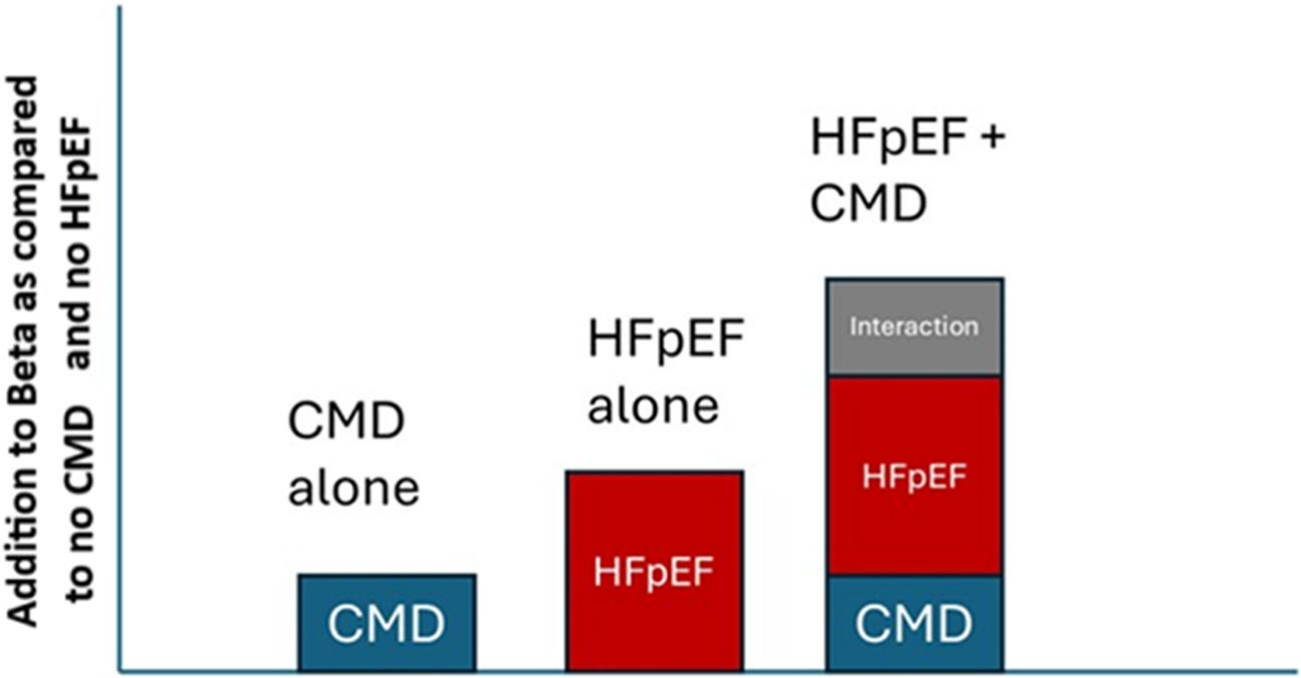
Additive effect of CMD and HFpEF on LV stiffness constant ß, The diagram below portrays the additive influence of HFpEF and CMD on LV stiffness constant, derived from the significant interaction between standardized beta coefficients of stiffness constant for both syndromes

CMD, but not the presence of HFpEF, was associated with systolic function as measured by GLS (HFpEF β* = 0.14; p = 0.09 vs. CMD β* = 0.31; *p* < 0.001) (Fig. 4). HFpEF had a numerically greater association than CMD with LARS (HFpEF β* = −0.29; *p* < 0.001 vs. CMD β* = −0.21; p = 0.02), E/E’ (β* = 0.44; *p* < 0.001 vs. β* = 0.29; *p* < 0.001). The estimated marginal means for the LV stiffness coefficient β showed a significant interaction between HFpEF and CMD (Fig. 5), demonstrating an additive and independent association of both CMD and HFpEF with the LV stiffness constant β (*p* = 0.044).

We performed a ROC analysis to identify whether GLS impairment was predictive of the presence of CMD in HFpEF patients. At a Youden-Index derived cut-off value for GLS of ≥ −17.55%, there was an odds ratio of 13.0 (95% CI [1.4–121], *p* = 0.02) for CMD to be present in HFpEF patients.

## Discussion

The main findings of our study can be summarized as follows:

1. Patients assessed for CMD have a high prevalence of HFpEF.
2. CMD is associated with changes in both systolic and diastolic function independently of a diagnosis of HFpEF.
3. CMD and HFpEF are independent and additive determinants of myocardial stiffness, suggesting a different pathophysiology and a multiplicative interaction.

HFpEF and CMD are complex, heterogeneous conditions with substantial epidemiological overlap, yet their pathophysiological interplay remains incompletely understood [6, 14, 15]. Our findings suggest that, while being associated with a high prevalence of HFpEF, CMD is an independent determinant of both systolic and diastolic function. These observations confirm the hypothesis that CMD might “drive myocardial dysfunction and remodelling” [16] in patients with HFpEF.

### Impact of CMD and HFpEF on diastolic function

As known for HFpEF [17], we demonstrate that also CMD, independently of HFpEF, is associated with adverse diastolic parameters, including elevated ventricular stiffness, reduced left atrial compliance, and higher estimated filling pressures.

Our finding that HFpEF and CMD have an additive effect on LV stiffness has important mechanistic implications and highlights the distinct, but complementary roles of both conditions in cardiac function. In HFpEF, stiffness is attributed to extracellular matrix remodeling, interstitial fibrosis, and energy-dependent impaired relaxation [18, 19]. CMD likely contributes to these phenomena by reducing the substrate bioavailability, leading to reduced activation of protein kinase G (PKG), diminished titin phosphorylation, and increased cardiomyocyte stiffness [20, 21]. The observed increase in the LV stiffness constant in the presence of both conditions, greater than the sum of individual effects, suggests complementary pathophysiological mechanisms with additive impact (Fig. 5).

Lastly, LARS reflects LA compliance and serves as an early marker of diastolic dysfunction, declining before overt dysfunction or LA enlargement [22, 23] in both CMD and HFpEF.

### Systolic dysfunction

A subclinical systolic dysfunction has been observed in some patients with HFpEF [24], and it has been shown to be an independent predictor of all-cause mortality and HF hospitalization [24, 25]. In our study, systolic dysfunction was only present in HFpEF patients with accompanying CMD, while patients without CMD had normal GLS values. This appears to suggest that CMD, but not HFpEF alone, is associated with the impaired systolic function and the related worse prognosis of HFpEF patients.

### Clinical implications

Our observations might have therapeutic implications. We demonstrate that CMD is the likely cause of subclinical impairments in systolic function in patients with HFpEF. We previously reported that reduced inotropic response, impaired ventriculo-arterial coupling, and higher extracellular volume fraction under stress describe a specific subset of patients that have characteristics of both HFpEF and HFrEF [17]. The current data are compatible with clinical evidence of a benefit of therapies affecting afterload in this HFpEF endotype [26–28]. We propose that targeting CMD with therapies aimed at improving microvascular perfusion may help preserve myocardial function and prevent progression to manifest heart failure. Given the interplay of CMD and HFpEF on myocardial stiffness, it is important to mention that newer medications in HFpEF such as SGLT2 inhibitors are thought to improve endothelial function and may therefore have the potential to mitigate CMD-related myocardial impairment [29].

## Limitation(s)

To minimize the confounding impact of markedly elevated LV filling pressures on CMD markers, we intentionally excluded patients with clinically advanced HFpEF. As a result, natriuretic peptide levels and echocardiographic signs of diastolic dysfunction were less pronounced than in typical HFpEF cohorts [8]. While this may limit generalizability to more severe HFpEF, it also allowed for the investigation of CMD in a well-characterized cohort with early or subclinical HFpEF features, potentially providing clearer insights into its independent relationship with myocardial stiffness.

We excluded patients with atrial fibrillation to minimize confounding from irregular rhythm on echocardiographic assessment of diastolic and systolic function, as well as on pressure–volume estimates. Therefore, our results are not generally transferable to patients with atrial fibrillation, which is a common comorbidity among patients with HFpEF [30].

Pressure–volume loop measurements were estimated from non-invasive imaging (echocardiography) and not through conductance catheters. However, this method has shown good correlation and validation in previous papers [13]. Lastly, by design, results from this study should be considered hypothesis-generating.

## Conclusion

CMD and HFpEF frequently coexist. Our findings highlight CMD as a key contributor to subtle systolic dysfunction, while both CMD and HFpEF impair diastolic function. HFpEF patients with coexisting CMD may thus represent a distinct phenotype that could potentially benefit from specific therapies, which warrants further investigation in future randomized trials.

## Data Availability

The data underlying this article will be shared on reasonable request to the corresponding author.

## Abbreviations

ANOCA: Angina with non-obstructive coronary artery disease
ATP: Adenosine Triphosphate
CFR: Coronary flow reserve
CMD: Coronary microvascular dysfunction
EDPVR: End-diastolic pressure volume relationship
GLS: Global longitudinal strain
IMR: Index of microvascular resistance
LARS: Left atrial reservoir strain
MRR: Microvascular resistance reserve
RRR: Resistive reserve ratio
